# Differential expression of miR-34b and androgen receptor pathway regulate prostate cancer aggressiveness between African-Americans and Caucasians

**DOI:** 10.18632/oncotarget.14198

**Published:** 2016-12-25

**Authors:** Marisa Shiina, Yutaka Hashimoto, Taku Kato, Soichiro Yamamura, Yuichiro Tanaka, Shahana Majid, Sharanjot Saini, Shahryari Varahram, Priyanka Kulkarni, Pritha Dasgupta, Yozo Mitsui, Mitsuho Sumida, Laura Tabatabai, Guoren Deng, Deepak Kumar, Rajvir Dahiya

**Affiliations:** ^1^ Department of Urology, Veterans Affairs Medical Center, San Francisco and University of California San Francisco, San Francisco, California, USA; ^2^ Division of Science and Mathematic, Cancer Research Laboratory, University of the District of Columbia, Washington, DC, USA

**Keywords:** miR-34b, prostate cancer, african-americans, caucasians, androgen receptor

## Abstract

African-Americans are diagnosed with more aggressive prostate cancers and have worse survival than Caucasians, however a comprehensive understanding of this health disparity remains unclear. To clarify the mechanisms leading to this disparity, we analyzed the potential involvement of miR-34b expression in African-Americans and Caucasians. miR-34b functions as a tumor suppressor and has a multi-functional role, through regulation of cell proliferation, cell cycle and apoptosis. We found that miR-34b expression is lower in human prostate cancer tissues from African-Americans compared to Caucasians. DNA hypermethylation of the miR-34b-3p promoter region showed significantly higher methylation in prostate cancer compared to normal samples. We then sequenced the promoter region of miR-34b-3p and found a chromosomal deletion in miR-34b in African-American prostate cancer cell line (MDA-PCA-2b) and not in Caucasian cell line (DU-145). We found that AR and ETV1 genes are differentially expressed in MDA-PCa-2b and DU-145 cells after overexpression of miR-34b. Direct interaction of miR-34b with the 3’ untranslated region of AR and ETV1 was validated by luciferase reporter assay. We found that miR-34b downregulation in African-Americans is inversely correlated with high AR levels that lead to increased cell proliferation. Overexpression of miR-34b in cell lines showed higher inhibition of cell proliferation, apoptosis and G1 arrest in the African-American cells (MDA-PCa-2b) compared to Caucasian cell line (DU-145). Taken together, our results show that differential expression of miR-34b and AR are associated with prostate cancer aggressiveness in African-Americans.

## INTRODUCTION

Prostate cancer (PCa) is the most commonly diagnosed cancer in American men and is the second leading cause of cancer mortality in the United States. African-Americans have the world's highest incidence of PCa and the mortality rate is more than two times greater than Caucasian men [1, 2]. This racial disparity has been attributed to differences in tumor growth rates, disease aggressiveness and genetic variants [3, 4]. Elucidating the mechanisms underlying these disparities is of crucial importance in reducing the incidence of prostate cancer in African-American males.

Androgen receptor (AR) signaling has been reported to have a critical role associated with prostate cancer in racial disparities [5, 6]. Studies show that AR expression was 22% higher in the benign prostate and 81% higher in prostate cancer of African-Americans compared to Caucasians [6]. The AR gene is located at Xq11.2-q12 (markers DXS991-DXS983) and is more than 90 kb in length. The amino-terminal domain is encoded by exon one, which includes highly polymorphic CAG repeats [7]. A previous study showed that Caucasian, African-American and Asian subjects predicted an increased risk of prostate cancer in men with short (≤21) CAG repeats [8]. Other studies found no association between the AR CAG repeat length and prostate cancer risk [9]. Evidence that AR CAG repeat length is associated with prostate cancer risk remains controversial [8]. ETV1 is overexpressed in 5–10% of prostate cancers [10]. Tomlins *et al*. [11] identified recurrent genomic rearrangements in prostate cancer resulting in the fusion of the 5′ untranslated end of *TMPRSS2* (a prostate-specific, androgen-responsive, transmembrane serine protease gene) to ETS family members (ERG, ETV1, ETV4). Studies show that ETS fusions are associated with a worse prognosis while other studies correlate with improved outcomes [5].

MicroRNAs (miRNAs) are 18–22 nucleotide noncoding regulatory RNAs which play a key regulatory role in gene expression at the posttranscriptional level [12]. In cancer, miRNA expression profiles have been found to be tissue type-specific and have been shown to be oncogenic or tumor-suppressors, implicating them as key regulators of cancer biology [13]. However, only a few reports exist in the literature describing the role of miRNAs in PCa aggressiveness and racial disparities [14]. miR-34b belongs to the miR-34 family of miRNAs: miR-34a, miR-34b, and miR-34c. miR-34b and miR-34c share a primary transcript on chromosome 11q23, whereas miR-34a is located at 1p36 and is encoded in its own transcript [15]. All of these miRNAs share the same seed sequence having similar endogenous mRNA targets. Promoter regions of mir-34a and mir-34b/c contain a match to the canonical p53 binding site and are direct p53 targets, which induce apoptosis, cell cycle arrest and senescence [16, 17]. miR-34b is a well-described tumor suppressor in a number of malignancies including colorectal, pancreatic, mammary, ovarian, urothelial, renal cell carcinomas and soft tissue sarcomas [18]. In the current study, we demonstrate that low miR-34b expression is responsible for aberrant expression of AR associated with prostate cancer progression and aggressiveness, especially among African-American men.

## RESULTS

### Lower expression of miR-34b in an African-American prostate cancer cell line and tissue samples compared to Caucasians

**Figure 1:**
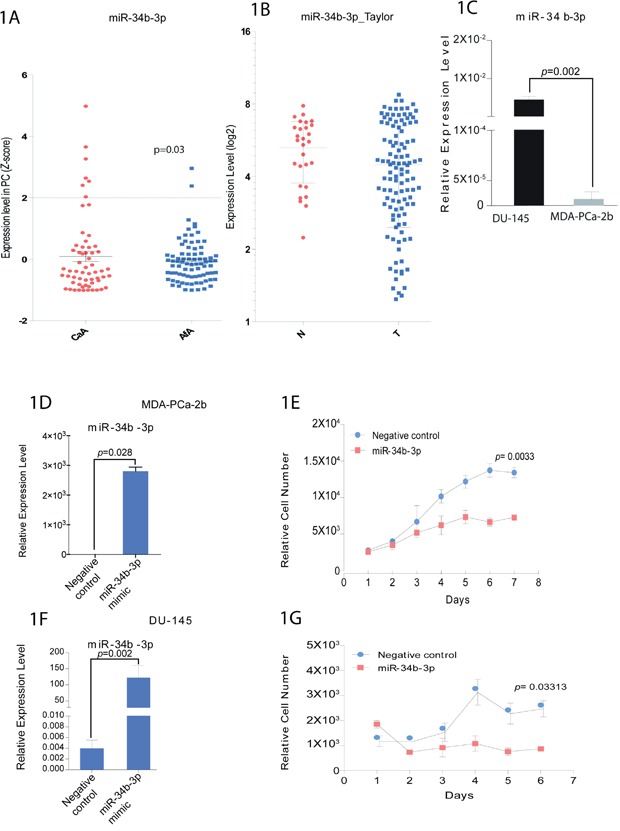
miR-34b expression in prostate cancer patients and cell viability in prostate cancer cell lines **A.** qPCR analysis for miR-34b-3p expression in Caucasians (CaA, n=62) and African-Americans (AfA, n=81) normalized by RNU48 (p<0.03). **B.** analysis of miR-34b levels on normal (N) and prostate tumor samples (T) from Taylor data. **C.** qPCR analysis for miR-34b-3p expression in DU-145 and MDA-PCa-2b cell lines. Bar=±SE. p-value was calculated by two-tailed t-test. **D** and **F.** MDA-PCa-2b cells or DU-145, respectively, were transfected with miR-34b-3p mimic for 72h and miR-34b-3p expression was evaluated by Taqman analysis. **E** and **G.** Cell viability for MDA-PCa-2b or DU-145, respectively, was measured using CellTiter-Glo assay and is shown as the relative cell number compared with control cells. Bar=±SE. p-value was calculated by two-tailed t-test.

To investigate if miR-34b expression could potentially be associated with biological differences between African-American and Caucasian prostate cancer, tumors samples were collected from 81 African-American and 62 Caucasian patients with localized disease. Clinicopathologic information is summarized in Supplemental Table 1. Expression analysis of miR-34b by qRT-PCR revealed that this miRNA was significantly correlated with race using Fisher's exact test (p=0.03) in African-American samples compared to Caucasian samples (Figure 1A). Also, analysis from Taylor data indicated that prostate tumor samples express lower level of miR-34b compared to normal samples (Figure 1B). In order to mimic the tissue sample results and help us identify mechanisms related to racial disparity, we selected two cell lines, DU-145 and MDA-PCa-2b, which express different levels of miR-34b. The African-American cell line, MDA-PCa-2b, expresses significantly lower amounts of miR-34b-3p compared to Caucasian cell line, DU-145 (Figure 1C).

### miR-34b overexpression decreases the cell viability of an African-American cell line more than Caucasian cell line

To determine the role played by miR-34b in differences between African-Americans and Caucasians, miR-34b was overexpressed in MDA-PCa-2b and DU-145 cells. We confirmed increased miR-34b expression by qRT-PCR after transfection of miR-34b-3p mimic (Figure 1D, 1F). We performed cell viability assays and found decreased cell viability in MDA-PCa-2b and DU-145 cells compared with negative control. Interestingly, the decrease in cell viability by miR-34b-3p mimic was more significant in MDA-PCa-2b (*p*=0.003) than DU-145 (*p*=0.03), suggesting that miR-34b has a more potent effect on the African-American cell line (Figure 1E, 1G).

### Chromosomal deletion in miR-34b in MDA-PCA-2b cell line

The Cancer Genome Atlas (TCGA) data portal (https://tcga-data.nci.nih.gov/tcga/) was used to validate differences in DNA hypermethylation of the miR-34b-3p promoter region and it showed significantly higher methylation in prostate cancer compared to normal samples (Supplemental Figure 1A). We then treated MDA-PCa-2b and DU-145 cell lines with 5-AZA-CdR and found no change in the expression of miR-34b in DU-145 or MDA-PCa-2b cells (Supplemental Figure 1B). We analyzed the relationship of miR-34b-3p chromosomal abnormality with the cBioPortal web tool for exome analysis data. These data showed that miR-34b was deleted in 3.4% of prostate adenocarcinoma patients (5/149) from the Nelson Lab at the Fred Hutchinson Cancer Research Center, 1.5% (5/332) of prostate adenocarcinomas from the TCGA database and 0.7% (1/150) of metastatic prostate cancers from a published article [20]. We then sequenced the promoter region of miR-34b-3p and found a chromosomal deletion in miR-34b in MDA-PCA-2b cells but not in DU-145 cells (Supplemental Figure 2).

### African-American and Caucasian prostate cancer cells have differences in apoptosis and cell cycle regulation

**Figure 2:**
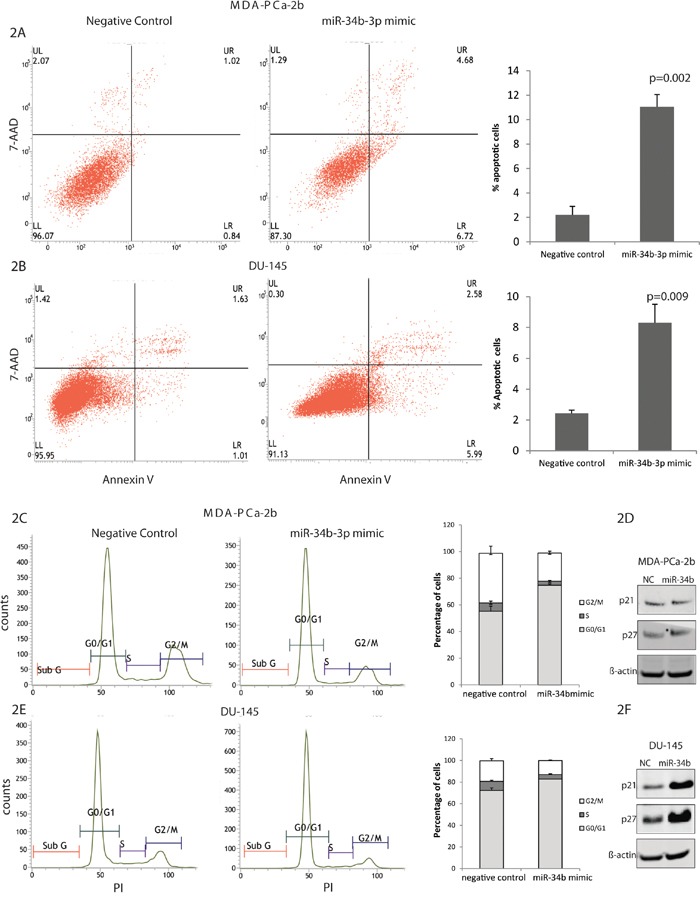
Effects of miR-34b overexpression on cell cycle and apoptosis of MDA-PCa-2b and DU-145 cells MDA-PCa- 2b and DU-145 cells were transfected with miR-34b mimic or negative control. **A** and **B.** Apoptosis in MDA-PCa-2b and DU-145 cells, respectively, were measured by flow cytometric analysis of cells labeled with Annexin-V/7AAD double staining. **C** and **D.** Cell cycle distribution of MDA-PCa-2b and DU-145 cells, respectively, was analyzed by propidium iodide staining by flow cytometry. **D** and **F.** Immunoblot analysis in MDA-PCa-2b and DU-145 cells, respectively, for p21 and p27 expression.

After overexpression of miR-34b-3p, the fraction of cells undergoing apoptosis was quantified by using Annexin V/7AAD staining. Both cell lines, MDA-PCa-2b and DU-145, transfected with miR-34b-3p showed a significantly increased apoptosis compared to negative control. DU-145 transfected cells showed 3 fold increases of apoptosis compared to control, while MDA-PCa-2b showed 5 fold increases of apoptosis, suggesting that miR-34b induces a higher apoptosis rate in African-American compared to Caucasian cells (Figure 2A-2B).

The cell cycle profile of MDA-PCa-2b and DU-145 cell lines overexpressed with miR-34b-3p showed an increase in the G1 phase (MDA-PCa-2b, NC 54.4% compared to miR-34b-3p transfected cells 74.7); (DU-145, NC 72.9% vs miR-34b-3p transfected cells 82.8%) suggesting that miR-34b-3p can induce G1 arrest in these cells lines (Figure 2C and 2E). Based on our Western blot results, DU-145 cells transfected with miR-34b-3p showed high expression of *p21*^Cip1^, *p27*^Kip1^, both involved in the regulation of cell cycle G1 arrest (Figure 2D). Interesting, MDA-PCa-2b cells transfected with miR-34b-3p did not show on increase in *p21*^Cip1^, *p27*^Kip1^ expression, suggesting that regulation of the cell cycle in these cells lines occurs by another mechanism (Figure 2F). Since MDA-PCa-2b cells have miR-34b-3p deletion, this may be a factor causing the difference in cell cycle regulation.

### PCR array analysis

**Figure 3:**
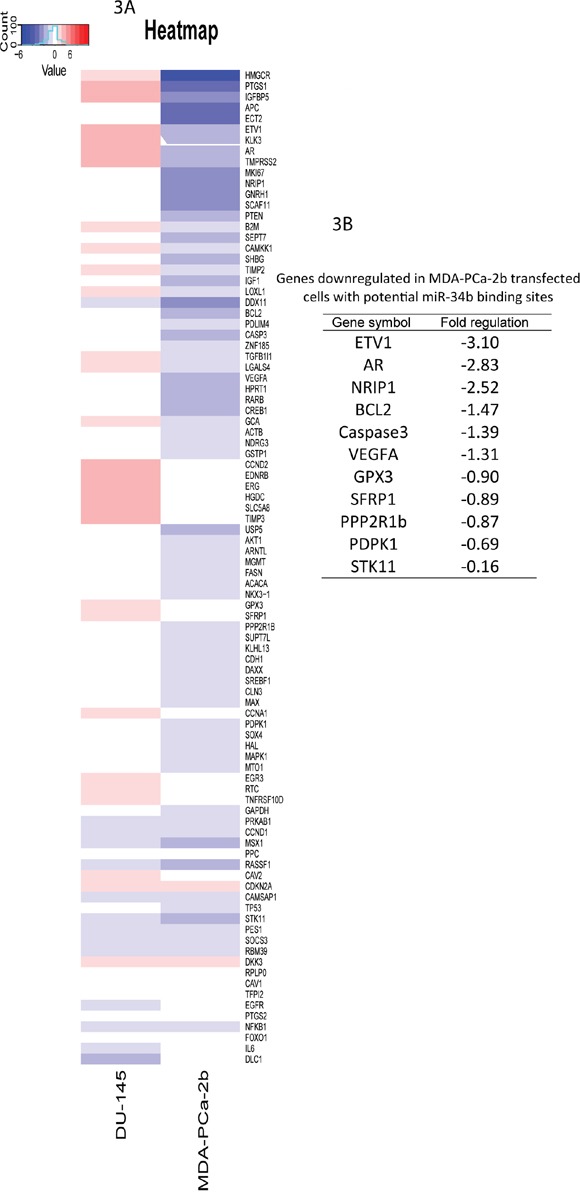
Gene expression changes in MDA-PCa-2b and DU-145 cells transfected with miR-34b-3p mimic was analyzed using RT2 Profiler PCR Array **A.** heatmap of genes with altered expression. Up-regulated genes are in red and down-regulated genes are in blue. **B.** down-regulated genes in MDA-PCa-2b upon miR-34b overexpression with potential miR-34b binding sites.

PCR array analysis was performed to determine the molecular effects of miR-34b overexpression in MDA-PCa-2b and DU-145 cells. Of the 84 genes represented in the Human Prostate Cancer RT^2^ Profiler PCR Array PAHS-135Z (Qiagen), heat map shows the expression levels of genes in MDA-PCA-2b or DU-145 cells after transfection with miR-34b-3p mimic (Figure 3A). These downregulated genes belong to 19 different pathways, including the androgen receptor and insulin signaling pathways (Table 1). From the genes downregulated in MDA-PCa-2b upon miR-34b transfection, we used miRwalk, TargetScan, miRanda and RNA22 to predict potential miR-34b binding sites and selected *AR*, *BcL2*, *ETV1, NRIP1, PDPK1, PPP2R1B, SCAF11* and *SFRP1* genes for further studies (Figure 3B).

**Table 1:** Pathway analysis for down-regulated genes in MDA-PCA-2b cells transfected with miR-34b-3p mimic

Pathway	size	candidates	p-value	q-value	source
Prostate cancer - Homo sapiens (human)	89	11 (12.4%)	1.E-13	7.E-11	KEGG
AMPK signaling pathway - Homo sapiens (human)	124	11 (8.9%)	5.E-12	1.E-09	KEGG
Pathways in cancer - Homo sapiens (human)	398	15 (3.8%)	1.E-10	2.E-08	KEGG
Integrated Pancreatic Cancer Pathway	170	11 (6.5%)	2.E-10	2.E-08	Wikipathways
Nongenotropic Androgen signaling	31	6 (19.4%)	4.E-09	5.E-07	PID
Coregulation of Androgen receptor activity	61	7 (11.5%)	8.E-09	8.E-07	PID
IGF signaling	36	6 (16.7%)	1.E-08	9.E-07	INOH
AMPK Signaling	68	7 (10.3%)	2.E-08	1.E-06	Wikipathways
SREBP signalling	68	7 (10.3%)	2.E-08	1.E-06	Wikipathways
Endometrial cancer - Homo sapiens (human)	52	6 (11.5%)	1.E-07	6.E-06	KEGG
Androgen receptor signaling pathway	89	7 (7.9%)	1.E-07	6.E-06	Wikipathways
mTOR signaling pathway - Homo sapiens (human)	60	6 (10.0%)	2.E-07	1.E-05	KEGG
Angiogenesis overview	61	6 (9.8%)	3.E-07	1.E-05	Wikipathways
Integrated Breast Cancer Pathway	64	6 (9.4%)	4.E-07	1.E-05	Wikipathways
human cytomegalovirus and map kinase pathways	16	4 (25.0%)	6.E-07	2.E-05	BioCarta
Ghrelin	39	5 (12.8%)	7.E-07	3.E-05	NetPath
insulin	78	6 (7.7%)	1.E-06	4.E-05	INOH
Interleukin-11 Signaling Pathway	44	5 (11.4%)	1.E-06	4.E-05	Wikipathways
insulin Mam	82	6 (7.3%)	2.E-06	5.E-05	INOH

### AR and ETV1 expression are regulated by miR-34b

**Figure 4:**
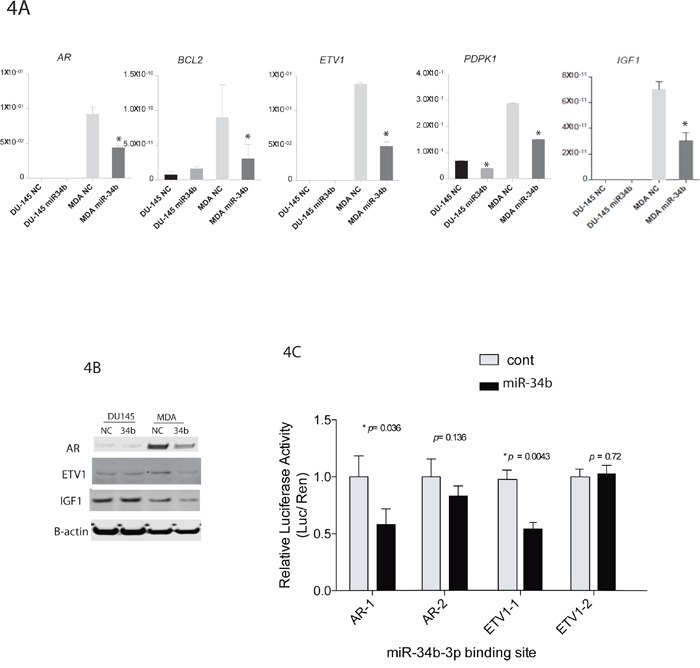
miR-34b target genes and luciferase reporter assay **A.** qPCR analysis of AR, BCL2, ETV1, PDPK1 and IGF1 from MDA-PCa-2b or DU-145 cells transfected with miR-34b-3p mimic. Asterisks indicate a p-value <0.05, calculated by two-tailed t-test. **B.** ETV1, IGF1 and AR levels were assessed by Western blot analysis. **C.** miR-34b binding sites in AR and ETV1 3′-UTR mediate the down-regulation of AR and ETV1 protein expression by miR-34b. p-value was calculated by two-tailed t-test.

We performed lentiviral vector-mediated expression of miR-34b in MDA-PCa-2b and DU-145 cells and subsequently we performed real-time PCR to validated differently expressed genes identified by PCR array analysis. Real-time PCR confirmed that 4 genes (AR, BcL2, ETV1 and PDPK1) with miR-34b binding site were downregulated in MDA-PCA-2b cells transfected with miR-34b mimic (Figure 4A). Western blot analysis showed that AR and ETV1 were significantly downregulated in miR-34b-3p MDA-PCa-2b transfected cells. Interestingly, miR-34b overexpression also caused a significant downregulation of IGF1 expression, at RNA and protein levels, in MDA-PCa-2b but not DU-145 cells (Figure 4A and 4B). Although IGF1 is not predicted to have a miR-34b binding site, IGF1 is closely related to the AR pathway and is one of the pathways that were significantly altered upon miR-34b overexpression in our pathway analysis (Table 1).

Also, luciferase reporter assay showed a significantly lower level of luciferase activity in miR-34b stably expressed HeLa cells when cells were transfected with the miR-34b binding site containing vectors, indicating a direct interaction between miR-34b and AR or ETV1 expression (Figure 4C).

### Level of AR inversely correlates with miR-34b

**Figure 5:**
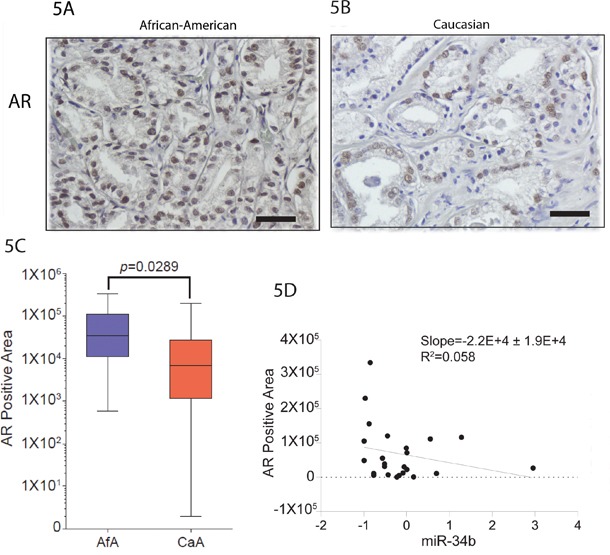
Staining pattern of AR in prostate cancer **A** and **B.** African-American and Caucasian tissue samples, respectively, were stained with AR. Original magnifications: × 200, scale bars: 500μm. **C.** Quantification of AR expression on African-American (Afa) and Caucasian (CaA) analyzed by IHC. **D.** Inverse correlation between the expression of AR protein analyzed by IHC and miR-34b analyzed by qPCR.

To determine whether our findings have clinical relevance, we examined the relationship between AR with miR-34b levels in the Caucasian and African-American FFPE samples by IHC. We found that protein level of AR is higher in African-American compared to Caucasian (Figure 5A and 5B). Interestingly, level of AR in African-American samples inversely correlates with miR-34b expression (Figure 5C). These indicate that our clinical data demonstrate that miR-34b correlates with AR expression and are associated with aggressiveness of prostate cancer in African-American males.

## DISCUSSION

It is well-known that miRNAs play a central role in the regulation of gene expression and miR-34b has been reported to be a tumor suppressor in many types of cancers [18, 21]. In this study, we provide novel insight into the role and regulation of miR-34b in African-American and Caucasian prostate cancer.

We report that miR-34b expression is lower in African-American prostate tumor samples compared to Caucasians. Analyzing 143 prostate tumors from 81 African-American and 62 Caucasian patients, we identified a significant loss of miR-34b in African-American compared to Caucasian samples (Figure 1). We also found that miR-34b directly controls transcription of AR and ETV1 leading to cell death.

The expression of miR-34b and miR-34c is low across all prostate cancers, due to allelic deletions and/or the loss of heterozygosity that frequently occurs at 11q23 [22]. Also, miR-34b can be epigenetically regulated through promoter hypermethylation in some prostate cancer cell lines and human tumor specimens. We found miR-34b chromosomal loss in MDA-PCa-2b but not in DU-145 cells (Supplemental Figure 2). Further analysis in additional tumor samples is necessary to fully elucidate the deletion of miR-34b in African-American patients.

In order to identify pathways and genes relevant to racial disparity, gene expression profiling analysis was performed using a prostate cancer pathway-focused PCR array with miR-34b transfected DU-145 and MDA-PCa-2b cells. Expression profiling of 84 genes showed that several genes were downregulated upon miR-34b overexpression in MDA-PCa-2b cells when compared to DU-145 transfected cells (Figure 3). Prediction based on TargetScan, miRwalk, RNA22 and miRanda analysis suggested that AR, BcL2, ETV1 and PDPK1 genes could be direct targets for miR-34b since it has a seed region able to bind to the 3’-UTR of these genes. Luciferase assay results clearly indicate that miR-34b regulates AR and ETV1 by direct binding to 3’-UTR of mRNA leading to translational repression (Figure 4C). The role of AR in the development and progression of prostate cancer has increased interest in this nuclear receptor [8]. Previous studies have shown that by integrating gene expression profiling and pathway analyses, multiple components within the AR signaling pathway have been shown to be upregulated in African-American prostate cancer [23]. Our PCR array analysis showed that AR was significantly downregulated in MDA-PCA-2b cells transfected with miR-34b compared with control. Although DU-145 cells, which express high levels of miR-34b, do not express AR, we examined the relationship between AR and miR-34b levels in Caucasian and African-American FFPE samples by IHC to determine whether our findings have clinical relevance. We found that AR protein levels are higher in African-American compared to Caucasian tissue samples (Figure 5A and 5B). Moreover, our study found low expression of miR-34b in African-American tissue samples compared to Caucasians, and its expression was inversely correlated with AR staining (Figure 1 and 5). This may explain the higher risk of aggressive prostate cancer in African-American men. In agreement, Yang *et al*. [24] suggested that AR-FL and AR-V7 activation mediated by hnRNPH1 in both a ligand-dependent and independent manner in African-Americans may confer prostate cancer progression. Also, it is reported that upregulation of PI3K/AKT, Wnt/B-catenin and IGF1 signaling pathways may contribute to activation of AR signaling and aggressiveness in African-American prostate cancer [23, 25]. Other studies show that IGF1 can activate AR by activating the PI3K/AKT pathway under low or absence of androgen levels [25–27]. Fan *et al*. [28] showed a positive feedback regulatory loop between AR and IGF1 that enhances AR activation, suggesting that pharmacological strategies that reduce IGF1 in combination with antiandrogen therapies may have clinical benefit in fighting prostate cancer. AR is a master regulator of the downstream androgen-dependent signaling pathway and suppresses the AKT pathway through FKBP5 and cyclin D1 leading to G1 cell cycle arrest [29, 30].

ETV1 is overexpressed in many prostate tumors and is associated with a higher Gleason score in aggressive prostate tumors [31–33]. Cai *et al*. [31] reported that ETV1 is an androgen receptor regulated gene that mediates prostate cancer cell invasion. Other studies show that ETV1 interacts and cooperates with AR signaling by favoring activation of the AR transcriptional program [5, 33]. Our study showed upregulation of AR in African-Americans when compared to Caucasian tissue samples (Figure 5A and 5B). We also observed inverse correlation of miR-34b and AR expression in our African-American tissue samples, suggesting that our clinical data demonstrate that miR-34b and AR are associated with aggressiveness of African-American prostate cancer (Figure 5C).

Importantly, the field of RNA therapeutics is currently undergoing a major expansion and miRNA-based therapies have already entered into clinical trials. miR-34 mimic (MRX34) has become the first microRNA to reach phase 1 clinical trials for hepatocellular carcinoma and chronic lymphocytic leukemia [34–37]. This phase 1 clinical trial represents an important step forward not only for miR-34 but is valuable proof of principle for the rationale of using miRNAs as anticancer drugs [37]. Further investigations are warranted to evaluate the potential of miR-34b in preclinical and clinical settings for prostate cancer and race disparities.

In summary, we have demonstrated that miR-34b expression is lower in African-American compared to Caucasian tissue samples and is inversely correlated with high AR level leading to cell proliferation and cancer progression. We concluded that miR-34b and AR play a pivotal role in the treatment of aggressive African-American prostate cancers.

## MATERIALS AND METHODS

### Patient samples and cell lines

Human prostate cell lines MDA-PCa-2b, DU-145 were obtained from the American Type Culture Collection. The Caucasian derived cell line, DU-145, was maintained in RPMI-1640 with 10% FBS and the African-American derived cell line, MDA-PCa-2b, was cultured in HPC1 with 20% FBS in Poly-L-Lysine (Sigma–Aldrich) coated culture dishes. Culture medium was supplemented with antibiotics and cells were cultured at 37°C with 5% CO_2_. African-Americans (n=41) and Caucasians (n=62) clinical FFPE (Formaldehyde Fixed Paraffin Embedded) samples were obtained from the Veterans Affair Medical Center, San Francisco, CA, USA. Additional African-American samples (n=40) were obtained from National Disease Research Interchange. Also, we used miRNA expression data from the Taylor data that is available at the Gene Expression Omnibus (GEO accession number: GSE21032).

### Transient transfection

In order to induce miR-34b-3p expression, cells were transfected with a mirVana miR-34b-3p Mimics (Thermo Fisher Scientific) using Lipofectamine RNAi Max (Thermo Fisher Scientific). To verify the transfection effect of miRNA mimics, mirVana miRNA Mimic Negative Control #1 (Thermo Fisher Scientific) was included in each transfection experiment.

### Cell cycle analysis

Transfected cells were harvested using Accutase (Corning), washed with cold PBS and fixed in cold 70% ethanol overnight at -20°C. Cell pellets were stained with PI/RNase Staining Buffer (BD Pharmingen) and incubated for 15 minutes at room temperature in the dark. Cells were analyzed for DNA content by gating excluding doublet cells on BD FACSVerse (BD Pharmingen).

### Apoptosis assay

Cells were transfected with miR-34b-3p mimic or negative control and harvested at different time points. Cells were washed in cold PBS, resuspended in 1x binding buffer and stained with Annexin V-FITC and 7AAD viability dye (Annexin V-FITC/7AAD kit, Beckman Coulter). After 15 minutes incubation at room temperature in the dark, cells were washed and analyzed using BD FACSVerse (BD Pharmingen).

### Western blot analysis

Cells were lysed with NP-40 (Thermo Scientific) plus Halt Protease and Phosphatase Inhibitor Cocktail (Thermo Scientific). Protein concentration was determined using BCA Protein Assay (Thermo Fisher Scientific). Western blots were performed using NuPAGE 4-12% Bis-Tris Protein Gels (Invitrogen). Gels were run in MES buffer (Invitrogen) and transferred onto nitrocellulose transfer membrane using iblot2 Dry Blotting System (Invitrogen). Membranes were incubated with Odyssey blocking buffer (Li-Cor) prior to incubation with primary antibodies overnight at 4°C. The following primary antibodies were used: AR (Cell Signaling, 5153), ETV1 (Thermo Fisher Scientific, PA5-41484), IGF1 (Thermo Fisher Scientific, PA5-27207), p21 (Cell Signaling, 2946), p27 (Cell Signaling, 2552), and ß-actin (Cell Signaling, 3700). Goat anti-rabbit IgG (H+L) 800 CW or goat anti-mouse (H+L) 680RD was applied for 45 minutes at room temperature (1: 15000, LI-COR) prior to washing with PBS containing Tween 20. Blots were imaged using an Odyssey Infrared Imaging System Scan and quantification was carried out with the LI-COR Odyssey® scanner and software (LI-COR Biosciences).

### Cell viability assay

Cell viability was determined using a *CellTiter*-*Glo* luminescent *cell viability* assay (Promega) according to the manufacturer's instructions. Measurements were performed every 24 hours for 6 days after transfections of miR-34b-3p mimics or negative control (Thermo Fisher Scientific) using a Victor X2 microplate reader (PerkinElmer).

### Quantitative real-time reverse transcription–polymerase chain reaction

Total RNA was isolated using a miRNeasy mini kit (Qiagen) and reverse-transcribed into cDNA with the SuperScript III kit (Life Technologies). Real-time reverse transcription–polymerase chain reaction (RT–PCR) was performed with SYBR Green (Applied Biosystems) using a Quant Studio 7 PCR System. Primer sequences are provided in Supplementary Table 2. For miR-34b-3p expression analysis, cDNA was synthesized from total RNA with TaqMan Reverse Transcription kit (Applied Biosystems) with specific primers and the cDNA was subjected to Taqman Probe-based Real Time PCR using and TaqMan miRNA assays Universal PCR Master Mix (Thermo Fisher Scientific) according to the manufacturer's instructions. The expression levels of miRNA were calculated as the amount of target miRNA relative to that of RNU48 control to normalize the initial input of total RNA.

### RT^2^ profiler PCR array analysis

Prostate cancer pathway-focused gene expression profiling was done using a 96-well human RT^2^ Profiler PCR Array PAHS-135Z (Qiagen). In this array, 84 genes were analyzed based on SYBR-Green real-time PCR. cDNA was synthesized from MDA-PCa-2b and DU-145 cells transfected with miR-34b-3p mimic using RT^2^ First Strand Kit (Qiagen) following the manufacturer's instructions. SYBR-Green real-time PCR was performed by following the manufacturer's instructions and fold-change calculations were accomplished using RT^2^ Profiler PCR Array Data Analysis (http://pcrdataanalysis.sabiosciences.com/pcr/arrayanalysis.php, Qiagen).

### Heat mapping and pathway analysis

All expression data obtained from RT^2^ Profiler PCR Array Data were calculated as the logarithm of each gene expression value and processed by global median centering normalization. Pathway mapping was done using R package “pathview” [19].

### Viral miRNA induction

Cells were infected with lentivirus containing either miR-34b-3p or empty in pmiR-lenti plasmid (Abm) to generate stable miR-34b-3p expression clones after growing with puromycin containing media (5μg/ml) for two weeks. Viral vectors were packaged by co-transfection into HEK293T9 cell line with pMD.2G and pPAX2 kindly obtained from Dr. Didier Trono.

### Immunohistochemical on human samples

Immunohistochemical (IHC) staining was performed in Caucasian and African-American prostate cancer specimens using Lab Vision™ UltraVision™ Detection System (Thermo Scientific). AR (Cell Signaling, 5153) was diluted 1:400 in 5% normal goat serum (Cell Signaling) and incubated overnight at 4°C. Brown color in DAB-stained IHC images were quantified using color deconvolution in ImageJ.

### Luciferase promoter assay and 3’-UTR luciferase reporter

For reporter assays, we used stable clones of HeLa cells that were established by viral infection with miR-34b or empty vector and transfected into each stable clone with miR-34b binding site containing luciferase reporter vectors. The sequences of the cloning primers are shown in Supplementary Table 2. Firefly luciferase activities were measured using Dual Luciferase Assay (Promega) after transfection and results normalized with Renilla luciferase.

## SUPPLEMENTARY MATERIALS FIGURES AND TABLES


