# Supplementary material for: Differential expression of miR-34b and androgen receptor pathway regulate prostate cancer aggressiveness between African-Americans and Caucasians
Source: Oncotarget. 2016 Dec 25;8(5):8356–68. doi: 10.18632/oncotarget.14198 (PMC5352406; doi:10.18632/oncotarget.14198)
Supplement: Supplementary file 1 [file oncotarget-08-8356-s001.pdf]

Differential expression of miR-34b and androgen receptor pathway regulate prostate cancer aggressiveness between African-Americans and Caucasians

SUPPLEMENTARY DATA

Drug treatment of cells

For demethylation studies, cells were treated daily with 10 µmol/L 5-Aza-Deoxycitydine(5Aza-CdR) (Sigma–Aldrich) for 72 hours.

In silico DNA methylation analysis

DNA hypermethylation of the miR-34b-3p promoter region was validated using TCGA data portal (https://tcga-data.nci.nih.gov/tcga/). The analyzed samples included 49 normal and 506 prostate cancer samples. We obtained TCGA DNA methylation data from the HumanMethylation 450 BeadChip (level 3).

Sequencing

Sequencing of the PCR product was performed to confirm genotyping. DNA was purified using a PCR purification kit (Qiagen). The ABI 377 Sequencer and Dye Terminator Cycle Sequencing kit (Applied Biosystem) was used to analyze the sequence of purified DNA products.

1A

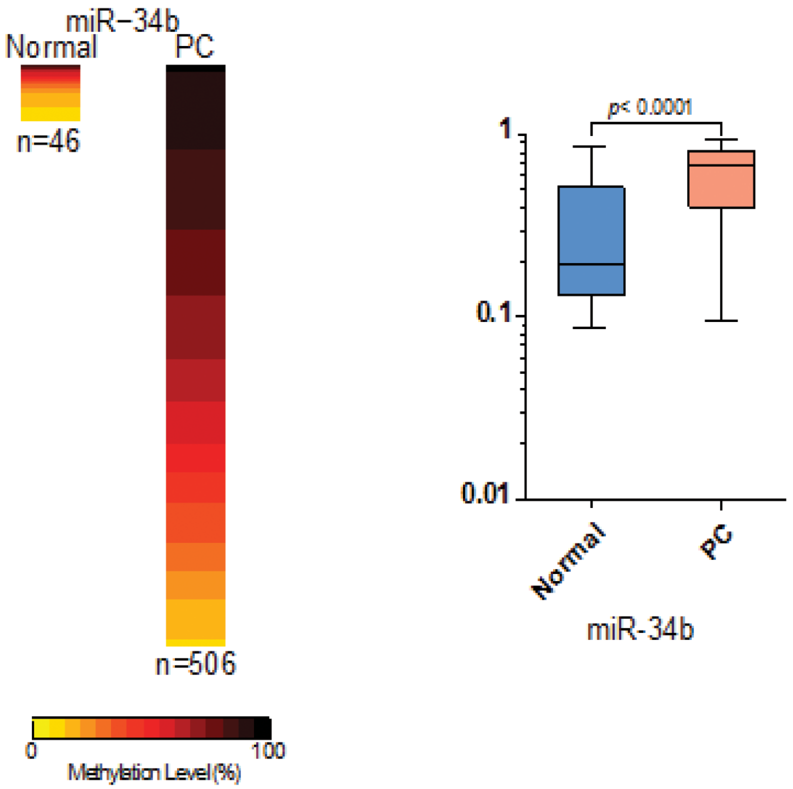

1B

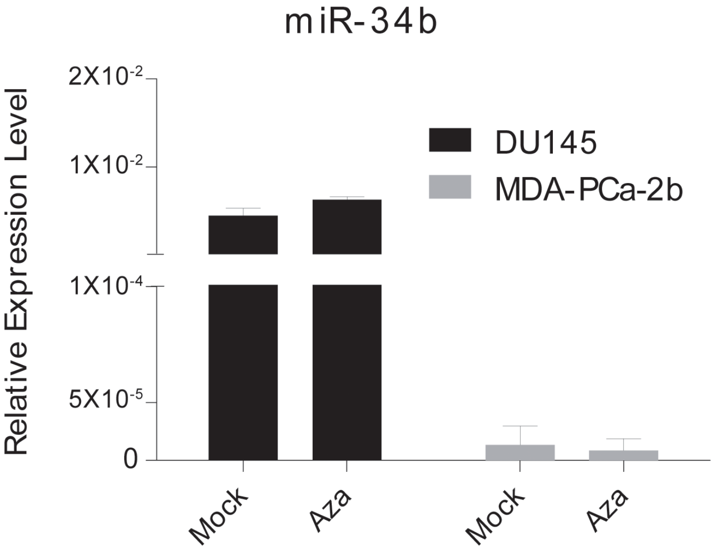

Supplementary Figure 1: A. methylation of miR-34b in normal tissue (n=46) and prostate tumors (n=506) data from TCGA cohort. **B.** qPCR analysis of miR-34-3p expression after 5-aza treatment for 72h in MDA-PCa-2b and DU-145 cell lines.

2A

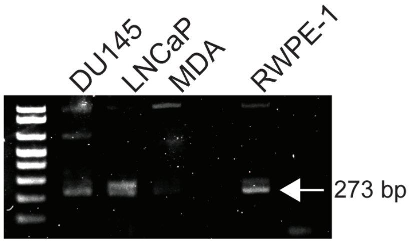

2B

62 °C 39cy, Phusion

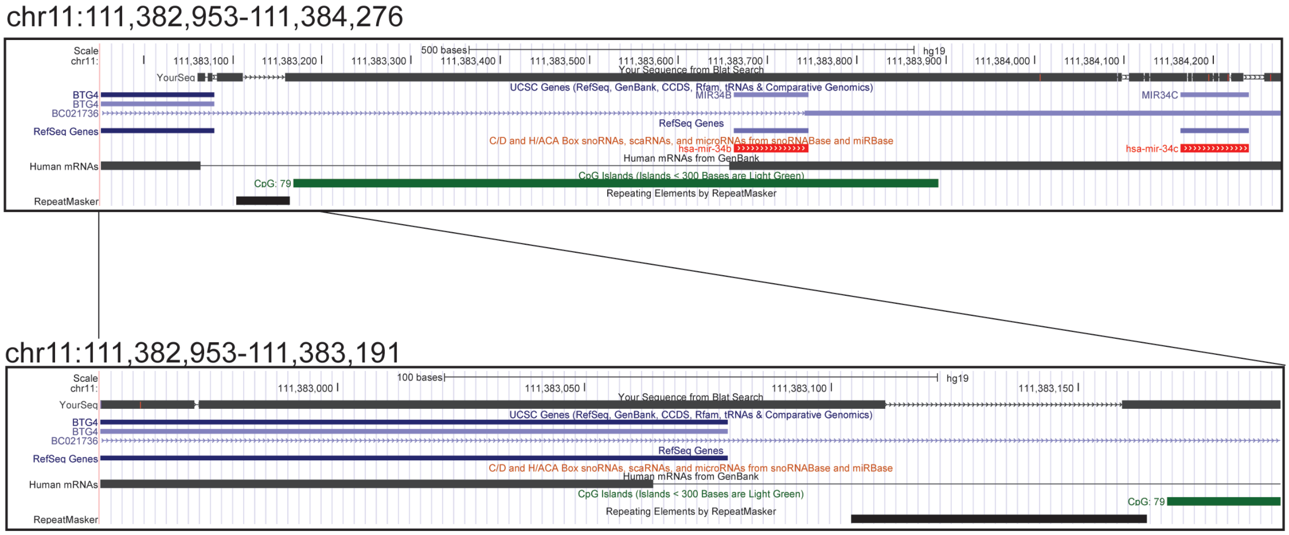

Sequence

DU-145

>CCGGAACAGCTGGGGGGCTTCTTGGGGATGAGGGAGTGGAGGAG
CTCTTTGTCCCTCCTGCTAGATCAGAAAGAGAAACGTCTCAAGAATCT
GGGCCTCCATCTTCTAGGCGTCTCCCTTGGAGGCCCTTCAGGGACC
GCCCACAGCGCTTTCTCTCAGCCTCCTCCCCTCGGCCCGCGGGGTT
CCAAGGACGGTTGGAAAGTCCCTTCCCCGGTGAAGAATTGGACTTCT
CTCTGCTTTCCCACCAACCCTGGAAAAACGGAGCCAGCAGTCGGGC
TAGAGCGGAAGCTGAGGGTCGCGGTTCCCAGGACGGTTGGGAGGA
AAAAAACTCCTCCCTCCTTCATTCCAAGAACGCCCCTCCCACCCTGT
TTCCGGGAAAATACCCCACGGGAAAAAAA

RWPE-1

>GCGGAGGTGCTGGGTGCTTCTTGGGGATGAGGGAGTGGAGGAGC
TCTTTGTCCCTCCTGCTAGATCAGAAAGAGAAACGTCTCAAGAATCTG
GGCCTCCATCTTCTAGGCGTCTCCCTTGGAGGCCCTTCAGGGACCG
CCCACAGCGCTTTCTCTCAGCCTCCTCCCCTCGGCCCGCGGGGTTC
CAAGGGACGGTTGGAAATTCGGGCCGAGCGCTGGACGGAAGAGAG
TTGGGGGGCCAACACCCCCCCCCGAGAAAAAAAACCCTTTTGCGGA
AGCGGAATCTTAGTGTCCTCGGGTTCTATAGACGGGAGGAAAGAAAA
TTATCTCTCCCCCCCCCAGCTCCCAGGAGGGCCCTTACCTTCTTGTT
TCCGTTTCAAATACACTTCGGACAAACT

Supplementary Figure 2: A. PCR product subjected to direct DNA sequencing. **B.** Sequencing alignment using UCSC browser.

Supplementary Table 1: Clinicopathologic characteristics of Caucasian and African-American prostate cancer patients

	Caucasian	African-American
	(n=62)	(n=41)
**Characteristic**	**N(%)**	**N(%)**
Age, years		
Median	62	61
Range	49-83	41-81
T-stage		
pT2	41 (66)	27 (66)
pT3-pT4	21 (34)	14 (34)
Gleason Score		
≥6	30 (48)	16 (39)
7 (3+4)	18 (29)	9 (22)
7 (4+3)	7 (11)	5 (12)
≤8	7 (11)	11 (27)
PSA failure		
Yes	22 (35)	20 (49)

Samples from the Veterans Affair Medical Center, San Francisco, CA, USA.

Supplementary Table 2: List of primers used for real-time PCR and luciferase assay

Primers	Sequence
qRT-PCR	
AR sense	GACGACCAGATGGCTGTCATT
AR antisense	GGGCGAAGTAGAGCATCCT
BCL2 sense	GGTGGGGTCATGTGTGTGG
BCL2 antisense	CGGTTCAGGTACTCAGTCATCC
ETV1 sense	CTGAACCCTGTAACTCCTTTCC
ETV1 antisense	AGACATCTGGCGTTGGTACATA
PDPK1 sense	TTCCGAGCTGGAAACGAGTAT
PDPK1 antisense	GGTCTCTTGCCTTAGGGAAGAA
IGF1 sense	GCTCTTCAGTTCGTGTGTGGA
IGF1 antisense	GCCTCCTTAGATCACAGCTCC
Luciferase assay	
miR-34b-AR S	AGCTTTGTTTAAACCCAAAAGCCTACCCAAGTGATTGTCTAGACTAG
miR-34b-AR AS	CTAGTCTAGACAATCACTTGGGTAGGCTTTTGGGTTTAAACAAATCT
miR-34b-AR S2	AAACATGGGTCCTTCACTAAGTGATTTT
miR-34b-AR AS2	CTAGAAAATCACTTAGTGAAGGACCCATGTTT
miR-34b-ETV1 S	AGCTTTGTTTAAACCTTATAAGGATGCTTGTGATTACTCTAGACTAG
miR-34b-ETV1 AS	CTAGTCTAGAGTAATCACAAGCATCCTTATAAGGTTTAAACAAAGCT
miR-34b-ETV1 S2	AGCTTTGTTTAAACATCTTATCATTTGCTAGTGATTATCTAGACTAG
miR-34b-ETV1 AS2	CTAGTCTAGATAATCACTAGCAAATGATAAGATGTTTAAACAAAGCT

AR, ETV1 and PDPK1 primer sequences for qRT-PCR were obtained from Harvard Primer Bank (http://pga.mgh.harvard.edu/*primerbank*/). Other primers were designed based on TargetScan, miRWalk and RNA22 database.
